# Individual and contextual determinants associated with bullying in schoolchildren eight to ten years of age

**DOI:** 10.1590/0103-6440202406084

**Published:** 2024-09-16

**Authors:** Luiza Jordânia Serafim de Araújo, Monalisa Cesarino Gomes, Ramon Targino Firmino, Edja Maria Melo de Brito Costa, Saul Martins de Paiva, Ana Flávia Granville-Garcia

**Affiliations:** 1 Department of Dentistry, School of Dentistry, Universidade Estadual da Paraíba, Campina Grande, PB, Brazil; 2 Department of Dentistry, Centro Universitário UNIFACISA, Campina Grande, PB, Brazil.; 3 Academic Unit of Biological Sciences, Universidade Federal de Campina Grande, Patos, PB, Brazil.; 4 Department of Pediatric Dentistry, Federal University of Minas Gerais, Minas Gerais, PB, Brazil.

**Keywords:** Bullying, anxiety, family relations, health education, Oral health, Bullying, Ansiedade, Relações familiares, Educação em saúde bucal, saúde bucal

## Abstract

Investigate individual and contextual determinants associated with bullying in schoolchildren eight to ten years of age. A cross-sectional study was conducted with 739 schoolchildren, who answered a question about episodes of bullying related to oral health and questionnaires addressing childhood anxiety and orofacial dysfunction. The guardians provided information on sociodemographic characteristics, sleep disorders, and oral health literacy. Trained examiners assessed the children for the diagnosis of dental caries using the International Caries Detection and Assessment System, malocclusion using the Dental Aesthetic Index, orofacial dysfunction using the Nordic Orofacial Test-Screening and traumatic dental injury (Andreasen criteria) (Kappa> 0.80). The contextual variables were the type of school and the monthly income of the school neighborhood. Descriptive statistics was performed to characterize the sample and unadjusted and adjusted (p <0.05) multilevel Poisson regression models were run. The prevalence of bullying was 13.3%. After the adjusted analysis, malocclusion (PR=1.59; 95%CI:1.03-2.44) and anxiety (PR=1.79; 95%CI:1.10-2.93) remained associated with bullying. In terms of context, the monthly income of the neighborhood of the school was associated with bullying (PR=1.75; 95%CI:1.12-2.72). Malocclusion and anxiety influenced the occurrence of bullying. A lower average income in the school neighborhood was an important contextual determinant for the increase in the prevalence of bullying.

## Resumo

Investigar determinantes individuais e contextuais associados ao bullying em escolares de 8 a 10 anos. Foi realizado estudo transversal com 739 crianças. As crianças responderam uma pergunta sobre episódios de bullying relacionado a saúde bucal, um questionário sobre ansiedade infantil e disfunção orofacial. Os responsáveis forneceram informações sobre características sociodemográficas, distúrbios do sono e alfabetismo em saúde bucal. Examinadores treinados avaliaram as crianças para diagnóstico da cárie dentária pelo *International Caries Detection and Assessment System*, má oclusão por meio do *Dental Aesthetic Index,* disfunção orofacial pelo *Nordic Orofacial Test-Screening* e traumatismo dentário (Critério de *Andreasen*) (Kappa>0,80). As variáveis contextuais foram tipo de escola e renda mensal do bairro escolar. Estatística descritiva foi utilizada para caracterização da amostra. Modelos de regressão de Poisson multinível não ajustados e ajustados (p<0,05) foram usados. A prevalência do bullying foi 13,3%. Após análise ajustada a má oclusão (RP=1.59; IC95%:1.03-2.44) e a ansiedade (RP=1.79; IC95%:1.10-2.93) permaneceram associados ao bullying. Em relação ao contexto, a menor renda mensal do bairro da escola foi associada ao bullying (RP=1.75; IC95%:1.12-2.72). Má oclusão e ansiedade influenciaram a presença do bullying. A menor renda mensal do bairro escolar foi um determinante contextual importante para o aumento da prevalência de bullying.

## Introduction

Bullying is defined as a public health problem that involves a set of repeated, purposeful, aggressive attitudes in a relationship of unequal power often present in the school-age period ^1^. Victims of bullying are prone to the emergence of psychosocial and physiological problems that can lead to low academic yield, truancy from school, serious mental health problems, such as anxiety and insecurity, as well as late-onset consequences, such as social exclusion ^2^.

Children experience episodes of bullying in the school phase, with variations in the prevalence related to sociocultural and economic aspects ^1^. One study reported that the prevalence of bullying related to oral health was 27% among children eight to ten years of age ^3^. Thus, oral problems, such as dental caries, traumatic dental injury, and malocclusion, are potential targets of bullying among children ^4^. Other problems, such as orofacial dysfunction, which is characterized as an imbalance in normal physiological processes, such as speech and facial expression, may trigger bullying in the school-age period ^5^.

A problem that has been a cause of concern among researchers is the oral health literacy of parents/guardians. This aspect could increase the prevalence of bullying in children, as low oral health literacy of parents/guardians is related to more oral health needs, poorer health outcomes in children, and difficulties making decisions in healthcare services, as children depend directly on their parents ^6^. Therefore, this is one of the hypotheses of the present investigation, as there are no studies that assess the possible association between the oral health literacy of parents and the occurrence of bullying in their children. Clarifying this association can contribute to increased awareness on the part of health professionals to take a holistic approach considering the possible role of oral health literacy in psychological aspects in children.

Problems related to sleep, an essential physiological process in the healthy development of physical and mental health ^7^, may also be associated with bullying in children. Insufficient sleep has become common among children and is associated with poor daily functioning, affecting behavioral and emotional aspects, making children and less productive ^7^. Nonetheless, sleep disorders have not yet been associated with bullying related to oral health. Studies addressing this issue can provide important data to enable targeted interventions.

As childhood is an important period for mental development, anxious children and those with low self-esteem may have difficulty reacting and making decisions ^8^. Emotions are more affected in this phase, which makes these children more vulnerable in the presence of classmates; anxiety is considered one of the most prevalent psychological problems in childhood ^8^. Thus, the level of childhood anxiety may also exert an influence on the occurrence of bullying in children. However, this aspect has not been explored or related to oral problems in schoolchildren eight to ten years of age.

Besides the factors mentioned, school context can exert a significant impact on the occurrence of bullying. The school environment is an essential setting for coexistence with peers and can become a place of risk for aggressive behavior ^9^. One study showed that public schools offer fewer prevention and health promotion activities ^10^. Thus, the type of school and the average income of the school neighborhood may influence the occurrence of bullying.

Therefore, the present study aimed to investigate individual and contextual determinants associated with bullying in schoolchildren eight to ten years of age in the city of Campina Grande in northeastern Brazil.

## Methods

### Ethics approval statement

This study received approval from the Human Research Ethics Committee of Universidade Estadual da Paraíba (approval nº 3.255.174; certificate of presentation: 10514619.2.0000.5187) in accordance with Resolution nº 466/2012 of the National Board of Health and followed the ethical principles established by the Declaration of Helsinki (2013). The children signed an assent form after their parents/caregivers signed a statement of informed consent.

### Study design and sample

A cross-sectional study was developed with 739 schoolchildren eight to ten years of age enrolled in public and private schools in the city of Campina Grande, Brazil. The sample was obtained through a probabilistic sampling procedure in two stages (schools and children) involving weighting and adjustment for the design effect. In the first stage, 14 public schools and 18 private schools distributed amongst the six geographical regions of the city were randomly selected. The minimum sample size was calculated considering the number of public and private schools in each geographical region. In the second stage, participants were randomly selected from each school through a simple random drawing based on attendance lists. The process resulted in a sample comprising 520 students from public schools and 249 from private schools. The sample size was calculated for an analytical study involving the comparison of two proportions using the G*Power program, version 3.1.9.7 (Franz Faul, Universitat Kiel, Germany), considering a 95% significance level and 95% study power. Based on the pilot study, the proportion of orofacial dysfunction among adolescents with and without bullying was 80% and 60%, respectively. Data on this variable provided the largest sample to investigate the association of interest. The minimum sample was calculated as 286 adolescents, to which a design effect of 2.0 was applied, and 25% was added to compensate for possible dropouts, leading to a target sample of 763 adolescents.

### Eligibility Criteria

Children of eight to ten years of age of both sexes enrolled in public and private schools in the city of Campina Grande were included. Children with developmental disorders or cognitive deficiency reported by the guardians, those submitted to past or present orthodontic treatment, those whose parents had a self-reported inability to read and speak Portuguese, and those with vision or hearing impairment were excluded from the study.

### Training and Calibration Exercises

Training took place in two steps. In the theoretical step, a specialist supervised the training of four dentists for the diagnosis of malocclusion using the Dental Aesthetic Index (DAI), dental caries using the International Caries Detection and Assessment System (ICDAS), orofacial dysfunction using the Nordic Orofacial Test-Screening (NOT-S) and traumatic dental injury using the criteria proposed by Andreasen et al. (2007). In the practical step, the examiners were asked to diagnose oral problems. A specialist in orthodontics and pediatric dentistry conducted the calibration exercise and served as the "gold standard" for inter-examiner agreements. This step was conducted with 40 children (20 from a public school and 20 from a private school) who were chosen by convenience. The children were reexamined after a seven-day interval to estimate intra-examiner agreement. The Kappa coefficients revealed good agreement (K > 0.80).

### Pilot Study

A pilot study was conducted with 40 school children randomly selected from the attendance list. The school was selected by convenience. The results of the pilot study revealed no need to alter the methods. These children were not included in the main study.

### Collection of Non-Clinical Data

Children were approached in the classroom, where the data collection process was explained. The informed consent form, sociodemographic questionnaire, Sleep Disturbance Scale for Children, and the Oral Health Literacy-Adults Questionnaire were sent home to be answered by parents/caregivers.

The sociodemographic questionnaire was composed of objective items related to the parent/guardian (monthly family income and level of schooling) and child (sex and age). These variables were collected as continuous data and dichotomized by the median.

Sleep disorders were investigated using the Sleep Disturbance Scale for Children (SDSC), which has been validated for children/adolescents three to 18 years of age. The SDSC is composed of 26 items that identify disorders of initiating and maintaining sleep, sleep breathing disorders, disorders of arousal, sleep-wake transition disorders, disorders of excessive somnolence, and sleep hyperhidrosis. The sum of the scores furnishes a total that ranges from 26 to 130 points. In the present study, a cutoff point of 39 was adopted to characterize the presence of sleep disorders ^11^.

Parents/guardians answered the Oral Health Literacy-Adults Questionnaire (OHL-AQ), which has 17 items addressing individual characteristics related to writing, reading comprehension, listening, numeracy, and decision-making related to oral health. Correct answers are assigned a score of 1 and incorrect answers are assigned a score of zero. The total is calculated from the sum of the individual items and ranges from 0 to 17 points, with higher scores denoting a higher level of oral health literacy (OHL) ^12^. For statistical purposes, the scores were divided into terciles: 0-11 = inadequate OHL; 12-13 = marginal OHL; and 14-17 = adequate OHL.

The children answered a question on bullying related to oral health was taken from a validated questionnaire (Child Perceptions Questionnaire 8-10). The examiners approached children in the classroom and asked whether other children ever teased or called the respondent names because of her or his teeth or mouth. Cronbach’s alpha for this question was 0.95 in the validation process of the questionnaire, which shows high reliability for use in the target population ^13^.

The children also answered a questionnaire addressing anxiety - the Revised Children’s Manifest Anxiety Scale (RCMAS), which is a psychometric instrument for the assessment of anxiety in children eight to 13 years of age. The RCMAS is composed of 37 items separated into two scales - one for the assessment of anxiety and one denominated the 'lie' scale. The index is obtained by the total sum of the components with ‘yes’ answers (assigned a score of 1) and ‘no’ answers (assigned a score of zero). The total ranges from 0 to 37, with higher scores denoting a greater trait of anxiety. Only the anxiety scale was used in the present study. For statistical purposes, the anxiety score was dichotomized by the cutoff point according to sex: anxiety was considered present when the score was ≥ 10.68 ± 5.32 for boys and ≥14.01 ± 5.57 for girls, as proposed in the study that validated the instrument in the Portuguese language ^14^.

Two variables were identified for the assessment of the contextual level of bullying: type of school (public or private) and average monthly income of the neighborhood of the school that the child attended. The type of school was collected on the first visit to the location and data referring to neighborhood income was obtained from the Brazilian Institute of Geography and Statistics ^15^

### Collection of Clinical Data

Prior to the clinical examinations, supervised toothbrushing was performed to facilitate the diagnosis, and the children were counseled about the importance of oral hygiene. Each child was examined in a reserved room at the school and stood in front of the examiner, who used personal protective equipment, an LED headlamp (Petzl Zoom), mouth mirror (PRISMA, SP, Brazil), Williams probe (WHO-621) and gauze.

The DAI was used for the diagnosis of malocclusion. This is a quantitative index to determine the degree of the aesthetic impact of malocclusion and orthodontic treatment needs: ≤ 25 = normal occlusion with no need for treatment; 26 to 30 = defined malocclusion suggesting elective treatment; 31 to 35 = severe malocclusion for which treatment is highly recommended; ≥ 36 very severe malocclusion for which treatment is mandatory ^16^. For statistical purposes, malocclusion was dichotomized as present (>25) or absent (≤ 25).

The ICDAS was used for the diagnosis of dental caries. This index scores carious lesions on a scale of 0 to 6 based on the extent of the lesion: 0 = sound; 1 = visible alteration after drying; 2 = visible color alteration after drying; 3 = enamel breakdown; 4 = dark shadow underlying dentin; 5 = cavity with dentin exposed at the base; and 6 = extensive cavity with dentin exposed at the base and on walls. Scores “1” and “2” were grouped into code 2, as it was not possible to use compressed air to dry the teeth ^17^. In the present study, cavitated dental caries on the anterior teeth were analyzed. Caries was dichotomized as absent (< 3) or present (≥ 3) on an anterior tooth.

The traumatic dental injury was investigated using the classification proposed by Andreasen et al. (2007): enamel fracture, enamel, and dentin fracture, complicated crown fracture, extrusive luxation, lateral, intrusive luxation, avulsion, discoloration and restoration due to injury ^18^. In the statistical analysis, the presence of any type of injury, color alteration, or restoration due to injury was considered.

Orofacial dysfunction was investigated with the aid of the Nordic Orofacial Test-Screening (NOT-S), which has been validated and adapted to Brazilian Portugues for use on children/adolescents eight to 14 years of age. The NOT-S has twelve domains in interview form (six domains) and a clinical examination (six domains). The interview addresses sensory function, breathing, habits, salivation, chewing, swallowing, and dry mouth sensations. The answers are dichotomized (yes or no). Each 'yes' answer is scored 1 point. The clinical examination section assesses respiration, face at rest, facial expression, masticatory muscles, and other mandibular functions as well as oral motor function and speech. Each item is classified as impaired or not impaired. Each impaired item is assigned 1 point. The total NOT-S score ranges from 0 to 12. Orofacial dysfunction was recorded when at least four domains had a 'yes' or 'impaired function' answer ^19^.

At the end of the clinical examination, topical fluoride was applied. Information on oral health status was given to the children and a letter was sent to the parents/guardians with orientations and referral to a primary care unit of reference if needed.

### Statistical Analysis

The data were organized and analyzed with the aid of SPSS Statistics (SPSS for Windows, version 25.0; IBM Inc., Armonk, NY, USA). Descriptive statistics were used for the characterization of the sample. Unadjusted and adjusted multilevel Poisson regression models were used to determine associations between the outcome variable and predictors. A random effects model was used for the multilevel Poisson regression analysis, with random intercepts and fixed slopes to investigate associations between bullying and individual/contextual covariables. This strategy enabled estimating prevalence ratios (PR) between comparison groups and respective 95% confidence intervals (CI).

In the first step, an unconditional (null) model was run to estimate the variability in the data before considering the individual and contextual characteristics. Covariables on the individual level were added in Model 2. Individual factors and contextual covariables were incorporated into the final model (Model 3). Individual variables with a p-value < 0.20 in the bivariate analysis were incorporated first and those with a p-value < 0.05 after the adjustments were maintained in Model 2. Next, contextual variables with a p-value < 0.20 in the bivariate analysis were incorporated, and those with a p-value < 0.05 after the adjustments were maintained in the final model. The goodness of fit of the models was calculated based on deviance values (-2 log-likelihood). Multicolinearity between variables was also checked.

## Results

The final sample was composed of 739 children, corresponding to a 97% response rate. The loss of data occurred due to three consecutive absences of students and refusal to participate in the study (3.9%).

Table 1 displays the characteristics of the sample. The prevalence of bullying was 13.3%. Caries on an anterior tooth were found in 25.8% of the children, traumatic dental injury was found in 16.2%, malocclusion was found in 49.1%, oral facial dysfunction was found in 33.3% and anxiety was found in 18.1%. Sleep disorder was found in 58.9% of the children and inadequate oral health literacy was found in 41.4% of parents/caregivers. The majority of the children attended private schools (52.8%) and the majority of schools were in neighborhoods with a higher average income (57%).

Table 2 displays the results of the multilevel bivariate Poisson regression analysis. In this step, bullying was associated with a lower level of guardian’s schooling (PR = 1.58; 95% CI: 1.01-2.49), dental caries on an anterior tooth (PR = 1.67; 95% CI: 1.04-2.65), orofacial dysfunction (PR = 1.89; 95% CI: 1.20-2.99), anxiety (PR = 1.76; 95% CI: 1.09-2.85), inadequate oral health literacy (PR = 1.88; 95% CI: 1.05-3.37) and lower average monthly income of the school neighborhood (PR = 1.89; 95% CI: 1.20-2.99).

The results of the multilevel multivariate Poisson regression analysis are displayed in Table 3. After adjustment for the individual variables (Model 2), the presence of caries on an anterior tooth (PR = 1.57; 95% CI: 1.03-2.39), malocclusion (PR = 1.62; 95% CI:1.05-2.49) and anxiety (PR = 1.93; 95% CI:1.18-3.14) were identified as individual determinants. In the final model (Model 3), the lower monthly income of the school neighborhood remained in the model after adjustment (PR = 1.75; 95% CI: 1.12-2.72). With regards to individual determinants after the adjustment of the context, dental caries on an anterior tooth lost its significance; only malocclusion (PR = 1.59; 95% CI: 1.03-2.44) and anxiety (PR = 1.79; 95% CI: 1.10-2.93) remained in the model.

**Table 1:** Characterization of the sample.

Variables	N (%)
Outcome variable	
Bullying	
No	640 (86.7)
Yes	99 (13.3)
Variables on an individual level	
Sex	
Male	370 (50.1)
Female	369 (49.9)
Monthly family income	
≤ US$ 235.84	327 (57.0)
> US$ 235.84	247 (43.0)
Guardian’s schooling	
≤ 8 years of study	318 (43.1)
> 8 years of study	419 (56.9)
Cavitated dental caries on anterior tooth	
No	548 (74.2)
Yes	191 (25.8)
Traumatic dental injury	
No	619 (83.8)
Yes	120 (16.2)
Malocclusion	
No	376 (50.9)
Yes	363 (49.1)
Orofacial dysfunction	
No	493 (66.7)
Yes	246 (33.3)
Mouth breathing	
No	698 (94.7)
Yes	39 (5.3)
Anxiety	
No	605 (81.9)
Yes	134 (18.1)
Oral health literacy	
Inadequate	306 (41.4)
Marginal	255 (34.5)
Adequate	178 (24.1)
Sleep disorder	
No	304 (41.1)
Yes	435 (58.9)
Variables on a contextual level	
Type of school	
Public	349 (47.2)
Private	390 (52.8)
Monthly income of school neighborhood	
≤ US$ 283.30	318 (43.0)
> US$ 283.30	421 (57.0)

**Table 2:** Bivariate analysis of the association between bullying and variables on individual and contextual levels among children eight to ten years of age.

Variable	Bullying		Bivariate (Unadjusted PR †)	
	Present n (%)	Absent n (%)	p-value	95% CI
Variables on an individual level				
Sex				
Male	52 (14.1)	317 (85.9)	0.390	1.22 (0.77-1.93)
Female	46 (12.5)	323 (87.5)		1.00
Monthly family income ^||^				
≤ US$ 235.84	46 (14.1)	281 (85.9)	0.111*	1.53 (0.90-2.61)
> US$ 235.84	30 (12.1)	217 (87.9)		1.00
Guardian’s schooling				
≤ 8 years of study	46 (14.8)	264 (85.2)	0.049*	1.58 (1.01-2.49)
> 8 years of study	52 (12.3)	372 (87.7)		1.00
Cavitated dental caries on anterior tooth				
No	64 (11.7)	483 (88.3)		1.00
Yes	34 (17.8)	157 (82.2)	0.031*	1.67 (1.04-2.65)
Traumatic dental injury				
No	78 (12.6)	540 (87.4)		1.00
Yes	20 (16.7)	100 (83.3)	0.162	1.45 (0.86-2.45)
Malocclusion				
No	43 (11.5)	332 (88.5)		1.00
Yes	55 (15.2)	308 (84.8)	0.076	1.53 (0.95-2.47)
Orofacial dysfunction				
No	49 (9.9)	444 (90.1)		1.00
Yes	49 (20.0)	196 (80.0)	0.006*	1.89 (1.20-2.99)
Mouth breathing				
No	91 (13.1)	606 (86.9)		1.00
Yes	7 (17.9)	32 (82.1)	0.396	1.42 (0.62-3.24)
Anxiety				
No	67 (11.1)	537 (88.9)		1.00
Yes	31 (23.1)	103 (76.9)	0.021*	1.76 (1.09-2.85)
Oral health literacy				
Inadequate	41 (16.1)	214 (83.9)	0.032*	1.88 (1.05-3.37)
Marginal	27 (15.3)	150 (84.7)	0.099	1.69 (0.90-3.19)
Adequate	30 (9.8)	276 (90.2)		1.00
Sleep disorder				
No	32 (10.5)	272 (89.5)		1.00
Yes	66 (15.2)	368 (84.8)	0.238	1.35 (0.81-2.26)
Variables on a contextual level				
Type of school				
Public	50 (14.4)	298 (85.6)	0.069*	1.54 (0.96-2.47)
Private	48 (12.3)	342 (87.7)		1.00
Monthly income of school neighborhood ^||^				
≤ US$ 283.30	56 (17.6)	262 (82.4)	0.006*	1.89 (1.20-2.99)
> US$ 283.30	42 (10.0)	378 (90.0)		1.00

†Unadjusted prevalence ratios (PR) for multilevel binomial regression to evaluate associations between individual/contextual variables and bullying among schoolchildren.

*Variables included in the multivariate model (p <0.20).

CI: confidence interval

**Table 3:** Multilevel Poisson regression for assessment of bullying according to variables on individual and contextual levels in children eight to ten years of age.

Fixed effects	Bullying		
	Model 1 (“Null”)	Model 2	Model 3
		PR (95% CI)	PR (95% CI)
Intercept	0.13 (0.11-0.16)	0.05 (0.03-0.09)	0.04 (0.02-0.08)
Variables on an individual level			
Monthly family income			
≤ US$ 235.84	-	-	-
> US$ 235.84	-	-	-
Guardian’s schooling			
≤ 8 years of study	-	-	-
> 8 years of study	-	-	-
Cavitated dental caries on anterior tooth			
No	-	1.00	-
Yes	-	1.57 (1.03-2.39)	-
Traumatic dental injury			
No	-	-	-
Yes	-	-	-
Malocclusion			
No	-	1.00	1.00
Yes	-	1.62 (1.05-2.49)	1.59 (1.03-2.44)
Orofacial dysfunction			
No	-	-	-
Yes	-	-	-
Anxiety			
No	-	1.00	1.00
Yes	-	1.93 (1.18-3.14)	1.79 (1.10-2.93)
Oral health literacy			
Inadequate	-	-	-
Marginal	-	-	-
Adequate	-	-	-
Variables on a contextual level			
Type of school			
Public	-	-	-
Private	-	-	-
Monthly income of school neighborhood			
≤ US$ 283.30	-	-	1.75 (1.12-2.72)
> US$ 283.30	-	-	1.00
Random effects			
Deviance (-2loglikelihood)	591,719	434,044	428,945

Model 1 (“null”): unconditional model; Model 2: individual variables; Model 3: variables on individual and contextual levels.

CI: confidence interval

## Discussion

In the present study, anxiety and malocclusion were associated with a greater frequency of bullying among children eight to ten years of age. A lower monthly income in the neighborhood where the school was located was the only contextual determinant associated with episodes of bullying. This study is relevant because it emphasizes a contextual analysis of the school environment and shows associations between bullying and anxiety, malocclusion, and monthly income of the school neighborhood in the eight to ten-year-old age group. The hypothesis that the oral health literacy of the parents/guardians would be associated with bullying was not confirmed, possibly because this is an indirect factor and does not exert a direct influence on bullying.

The prevalence of bullying was 13.3%, which is lower than the rate found in a previous study with a similar age range (eight to 10 years) ^3^. This difference may be due to differences in the populations studied. Regardless of this aspect, the prevalence found in the present investigation was sufficient to underscore the need for measures to avoid bullying and minimize the possible functional and psychosocial impacts throughout life.

The present findings suggest that anxiety is a risk factor for bullying. This aspect is a cause for concern, as anxious children do not have adequate social skills to deal with the problem and are prone to withdrawal and social isolation, which increases their vulnerability to bullying ^20^ ^,^ ^21^. A previous study with a broader age range found that anxiety can be a triggering factor for bullying and pointed out the bidirectional possibility of the association ^22^. Thus, the early identification of this condition and referral for psychological therapy could help children deal with anxiety and possibly reduce episodes of bullying at school.

Children with malocclusions suffered more episodes of bullying in this study. Malocclusion due to the exchange of dentitions can have aesthetic and functional impacts on children who are still in development and may trigger bullying ^4^ ^,^ ^23^. Thus, this study serves as a warning - children in this age group seem to have greater sensitivity to issues of dental aesthetics. Orientations for parents/guardians and the early treatment of cases of early malocclusion are needed not only to minimize functional and aesthetic problems but also possible episodes of bullying, which can exert an impact on the mental health of children. Likewise, it is essential to reinforce oral hygiene instructions and recommend regular visits to the dentist to avoid the development of malocclusion.

The presence of caries on anterior teeth was expected to remain associated with bullying, but this did not occur, probably because the age group studied was very close to the exchange of dentitions and there was likely not enough time for the formation of caries with greater decay on anterior teeth. In contrast, a previous study conducted with adolescents 11 to 14 years of age found an association between untreated caries and episodes of bullying ^24^.

In terms of context, it seems that the type of school was less important than the monthly income of the neighborhood where the school was located, possibly because the neighborhood environment exerts a greater influence on the attitudes of children than the type of school. A previous study found that children who live in an environment of economic deprivation may be more vulnerable to episodes of bullying ^25^. Educational campaigns should concentrate on environmental and behavioral factors, focusing on social disparities in the occurrence of bullying.

This study has limitations that should be considered. The cross-sectional design does not enable the establishment of cause-and-effect relationships between variables. Among the strong points, a pilot study was conducted to determine the viability of the methods and the examiners had undergone training and calibration exercises, minimizing the risk of bias. Another strong point was the representative sample, strengthening the external validity of the results. The present data are important to guiding policies directed not only at individual factors but also environmental factors.

In conclusion, anxiety and malocclusion are individual factors that exert an influence on the occurrence of bullying. In terms of context, a lower income in the neighborhood of the school that the children attended was associated with bullying.
